# MiR-192靶向负调控Bim表达诱导肺癌顺铂耐药

**DOI:** 10.3779/j.issn.1009-3419.2014.05.04

**Published:** 2014-05-20

**Authors:** 芳 张, 洋 李, 蘅 吴, 康 齐, 嘉琮 尤, 雪冰 李, 玲玲 祖, 振华 潘, 玉丽 王, 永文 李, 颖 李, 珉 王, 旺 沈, 清华 周

**Affiliations:** 300052 天津，天津市肺癌转移与肿瘤微环境重点实验室，天津市肺癌研究所，天津医科大学总医院 Tianjin Key Laboratory of Lung Cancer Metastasis and Tumor Microenvironment, Tianjin Lung Cancer Institute, Tianjin Medical University General Hospital, Tianjin 300052, China

**Keywords:** 肺肿瘤, miRNA芯片, RT-PCR, miR-192, Bim, Lung neoplasms, miRNA microarray, RT-PCR, miR-192, Bim

## Abstract

**背景与目的:**

顺铂是非小细胞肺癌化疗的一线药物，但获得性耐药限制了其疗效的发挥。本研究的目的是筛选鉴定与肺癌顺铂耐药相关的microRNAs，探讨其与肺癌顺铂耐药的影响及分子机制。

**方法:**

应用miRNA芯片及RT-PCR筛选鉴定A549与A549DDP肺癌细胞间的差异表达miRNAs，将差异表达miR-192转染A549和A549DDP细胞株，CCK-8检测miR-192对半数抑制浓度（half inhibition concentration, IC_50_）的影响，流式细胞术检测细胞凋亡，生物软件预测及双荧光素酶报告基因法寻找miR-192的靶基因，RT-PCR及Western blot检测靶基因表达水平在转染前后的变化。

**结果:**

MiR-192在A549DDP中显著高表达，表达量是A549细胞表达量的37.59±0.35倍。在低表达miR-192的A549细胞中过表达miR-192，细胞对顺铂的IC_50_显著增高，顺铂引起的细胞凋亡率显著降低；反之，在高表达miR-192的A549DDP中抑制miR-192的表达，顺铂的IC_50_显著降低，顺铂引起的细胞凋亡率显著增加。miR-192可靶向作用促凋亡基因*Bim*的3’-UTR，并在转录后水平负向调控Bim的表达。

**结论:**

MiR-192通过靶向负调控促凋亡基因*Bim*表达，诱导肺腺癌细胞株A549产生顺铂耐药，并减少顺铂引起的细胞凋亡。

## Abstract (en)

**Background and objective:**

Cisplatin is the first-line drug for the chemotherapy of non-small cell lung cancer (NSCLC), but the acquired chemoresistance restricted the effect of its treatment. The aim of this study is to validate the miRNAs related to the Cisplatin resistance in lung cancer and elucidate the molecular mechanisms.

**Methods:**

We performed miRNA microarray and RT-PCR to obtain the aberrant differential expressed miRNAs between A549 and its paired Cisplatin-resistant cell line A549/DDP cells, and then we investigated the biological functions of miR-192, which is the aberrant differential expressed miRNA. After transfection of the miR-192 into A549 cells, we measured the half inhibition concentration (IC_50_), cell apoptosis of the trasfectant cells, and then we used biological softwares and dual-luciferase report assay to explore the target gene of the miR-192, which was further validated by RT-PCR and Western blot.

**Result:**

MiR-192 was highly over-expressed in A549/DDP cells, whose quantity was 37.59±0.35 fold higher than that in A549 cells. Overexpression of miR-192 in A549 cells significantly conferred resistance to Cisplatin and inhibited apoptosis. By contrast, down-expression of miR-192 in A549/DDP cells remarkably restrained the Cisplatin resistance and induced apoptosis. MiR-192 binded to Bim 3'-UTR and negatively regulated Bim expression at the post-transcriptional level in lung adenocarcinoma cells.

**Conclusion:**

Our data suggested that miR-192 induced Cisplatin-resistance and inhibited cell apoptosis in lung cancer via negative targeting Bim expression.

肺癌是发病率和死亡率增长最快、对人类健康和生命威胁最大的恶性肿瘤之一，且仍然呈不断上升趋势^[1]^。化疗在肺癌的综合治疗中占有重要地位，但肿瘤对化疗药物产生耐药常常导致化疗失败，也是肿瘤复发、转移的主要原因。顺铂是非小细胞肺癌晚期治疗的一线化疗药物，但顺铂耐药是影响其疗效的主要障碍之一，因此克服和干预肿瘤细胞耐药是临床亟待解决的科学问题。

微小RNA（microRNA, miRNA）是生物体内源性长度约为22个-25个核苷酸的非编码小RNA，通过靶向作用于蛋白编码基因的3’-UTR区，影响目的基因的表达，从而影响细胞的生长、增殖、分化、凋亡等生物学行为^[2]^。研究^[3, 4]^报道证实miRNA可以发挥癌基因或者抑癌基因的功能，在肿瘤细胞的诊断、分期、进展、侵袭、预后等都发挥着重要的作用。迄今，有关miRNA在肺癌细胞顺铂耐药过程中所发挥的作用及其分子机制仍不清楚。

本研究选取肺腺癌细胞系A549及其耐顺铂细胞系A549/DDP作为研究对象，通过miRNA芯片等筛选出可能与肺腺癌耐药相关的miRNA，并探讨miRNA在肺癌细胞化疗耐药过程中所起的作用及其分子机制，以期能为逆转肺癌耐药提供理论基础和实验依据。

## 1 材料和方法

### 1.1 材料

人肺腺癌A549和A549/DDP细胞株由本实验室提供；顺铂、维拉帕米购自Sigma公司；Cell counting kit-8（CCK-8）购自DOjinDO公司；miRNeasy mini-Kit购自Qiagen公司；MiR-192 mimic和miR-192 inhibitor购自GenePharma公司；Lipofectamine^®^ 2000 Transfection Reagent购自Life Technologies公司；Hoechst33342和PI购自BD公司；FITC/Annexin V凋亡检测试剂盒购自BD公司；双荧光素酶报告基因载体pMIR-GLO和双荧光素酶报告分析试剂盒购自Promega公司；质粒小提试剂盒及胶回收试剂盒购自Axygen；TaKaRa MutanBEST Kit，感受态细胞（*E. coli* DH5α Competent Cells），限制性内切酶（*Sac*I、*Xba*I），M-MLV逆转录酶，实时荧光定量PCR试剂盒购自Takara；质粒测序由北京华大基因公司完成；蛋白裂解液RIPA购自碧云天公司；兔抗人Bim单抗购自Abcam公司。

### 1.2 方法

#### 1.2.1 MiRNA芯片及Real-time PCR筛选miRNA

收集A549细胞和A549/DDP细胞，miRNeasy mini-Kit抽提RNA，紫外分光光度仪和变性琼脂糖凝胶电泳法对RNA进行质检，将RNA送与上海博豪公司进行miRNA芯片杂交检测，将检测结果进行图像分析扫描及统计学分析，筛选出表达差异较为明显的miRNA（差异倍数在2倍及以上）。用miRNeasy mini-Kit提取小分子RNA，用SYBR^®^ Premix Ex Taq™逆转录合成cDNA，应用ABI Prism 7900HT实时定量系统进行PCR定量检测。MiRNA特异性的RT引物和PCR上下游引物序列如表 1所示。反转录条件: 16℃、30 min，42℃、60 min，85℃、5 min，4℃终止反应；PCR条件：95℃、30 s，一个循环；95℃、5 s，60℃、30 s，40个循环；95℃、15 s，60℃、1 min，95℃、15 s，一个循环。每管10 µL体系，设置3个平行样，U6作为内参。样本经过3次独立重复实验，所得数据使用比较CT值法（2^-ΔΔCt^）进行定量分析。样品目的基因的相对表达率（relative expression, RQ）采用∆∆CT方法计算，RQ =2^-ΔΔCt^（∆CT sample = CTsample - CTU6 sample, ∆CT control = CT control - CTU6 control, ∆∆CT = ∆CT sample - ∆CT control）。

**1:** 引物序列 Sequence of primers

Genes	Primers	Sequence (5’-3’)
miR-192	RT primer	GTCGTATCCAGTGCAGGGTCCGAGGTAT-TCGCACTGGATACGACGGGCTGT
	Forward primer Reverse primer	GGGGCTGACCTATGAATTGA CAGTGCAGGGTCCGAGGT
U6	RT primer	AACGCTTCACGAATTTGCGT
	Forward primer Reverse primer	CTCGCTTCGGCAGCACA CAGTGCAGGGTCCGAGGT

#### 1.2.2 细胞转染

将处于对数生长期的A549和A549/DDP细胞接种于6孔板中，当细胞密度达到40%-50%时，应用lipofectamin 2000转染试剂将miR-192 mimic、miR-192 inhibitor及各自的对照（negative control, NC）分别转染到A549和A549/DDP细胞中，并设置各自的空白对照（vehicle）组。6 h后换成完全培养基继续培养，转染24 h后检测转染效率。转染序列如下：miR-192 mimic：sense 5’-CUGACCUAUGAAUUGACAGCC-3’，anti-sense 5’-CUGUCAAUUCAUAGGUCAGUU-3’；mimic的NC：sense 5’-UUCUCCGAACGUGUCACGUTT-3’，anti-sense 5’-ACGUGACACGUUCGGAGAATT-3’；miR-192 inhibitor：5’-GGCUGUCAAUUCAUAGGUCAG-3’；inhibitor的NC：5’-CAGUACUUUUGUGUAGUACAA-3’。

#### 1.2.3 CCK-8检测半数抑制浓度（half inhibition concentration, IC_50_）

转染24 h后，0.25%胰酶消化细胞，制成5×10^4^/mL单细胞悬液，将细胞接种于96孔板，每孔100 μL（即5, 000个细胞/孔）。接种24 h，待细胞贴壁且恢复生长活性后，弃去培养基，加入含顺铂终浓度为0 μg/mL、1 μg/mL、2 μg/mL、4 μg/mL、8 μg/mL、16 μg/mL、32 μg/mL、64 μg/mL RPMI-1640培养基，每孔100 μL，每个浓度设置5个复孔。继续培养48 h后，每孔加入CCK-8溶液10 μL，轻轻振荡混合均匀，在37℃培养箱中继续培养1 h后，在酶联免疫吸附仪上采用450 nm波长测量每孔吸光度（optical density, OD）值。取5个孔的平均吸光度值，重复5次独立实验后取平均值。根据数据绘制细胞生长抑制率曲线，并计算顺铂对细胞的IC_50_。抑制率计算公式为：生长抑制率=（1-OD_用药组_/OD_对照组_）×100%，以最小二乘法进行曲线拟合，得到IC_50_值。

#### 1.2.4 流式细胞术检测细胞凋亡

转染24 h后，A549细胞6孔板每孔加入终浓度为2 μg/mL的顺铂，A549/DDP细胞每孔加入终浓度为8 μg/mL的顺铂。继续培养48 h后，1×PBS洗两遍细胞，0.25%胰酶消化，用10% RPMI-1640终止消化后，将细胞收集到EP管中，1, 000 rpm，4℃离心5 min后，去上清，用1×PBS洗两遍后，用FITC/Annexin V凋亡检测试剂盒进行染色，首先，每个样本中加入500 μL 1×buffer，吹打成单细胞悬液，后加入AnnexinV和PI各5 μL，37℃避光染色20 min后，用FACSAria™流式细胞检测染色细胞，WinMDI 2.9软件进行分析。

#### 1.2.5 双荧光素酶报告基因

通过miRanda、TargetScan、PicTar和RNA22等生物学软件预测Bim为miR-192的靶基因之一。通过PCR扩增miR-192作用于Bim的3’-UTR区，同时用TaKaRa MutanBEST Kit突变靶基因Bim 3’-UTR区中的miR-192的靶点，使miR-192与Bim脱靶，通过质粒构建的方法，将含miR-192作用靶点的Bim 3’-UTR区和已经将miR-192作用靶点突变的mutant-Bim3’-UTR区连接到双荧光素报告质粒pMIR-GLO的*Sac*I、*Xba*I两酶切位点之间，构建双荧光素酶报告基因质粒。构建的含Bim 3’-UTR和mutant-Bim3’-UTR的质粒送华大基因测序。构建成功的质粒转化感受态大肠杆菌DH5α，转化产物涂于含氨苄青霉素（Amp）的LB琼脂板上，37℃培养过夜。待LB琼脂板长出菌落后，挑取单菌落至于液体LB培养基中摇菌扩增。应用质粒小提试剂盒提取质粒，测定质粒浓度后，进行细胞转染。扩增Bim 3’-UTR的上游引物序列为5’-GGGGAGCTCGCAATAAACACACACAAAATAG-3’，下游引物序列为5’-GCCTCTAGACACATCACACAGAAAAAGAATC-3’，构建mutant-Bim 3’-UTR的上游引物序列为5’-TTGCAGATATTACTTATCAACTGAGCCAAAT-3’，下游引物序列为5’- CGAATCTAATAAATACTCACAATATATAC-3’。取对数生长的细胞接种48孔板，24 h后进行质粒转染，按照lipofectamine 2000说明书进行转染，每孔加入250 μL无血清培养基，培养基中含终浓度为1 μg/mL的pMIR-GLO质粒，0.2 μg/mL的pRL-SV40质粒，100 nmol/L的miR-192 mimic或miR-192 inhibitor。在转染后48 h，用Promega公司的双报告分析系统按说明书来测量海肾萤光素酶活性。

#### 1.2.6 Real-time PCR和Western blot检测细胞转染后Bim表达水平

MiR-192 mimic和miR-192 inhibitor分别转染A549和A549/DDP细胞48 h后，收集细胞，提取RNA，用RT-PCR检测转染前后细胞内*Bim*基因mRNA水平的变化。同时，转染48 h后，收集细胞，加入RIPA裂解细胞，提取总蛋白，BCA试剂盒进行蛋白定量，进行SDS-PAGE电泳，将电泳分离的蛋白电转移至NC膜上，5%脱脂牛奶封闭，分别加入1:2, 000兔抗人Bim抗体，4℃过夜孵育，TBST洗3次，每次10 min。加入辣根过氧化物酶偶联的抗兔（1:2, 000稀释）二抗，37℃，孵育1 h，TBST缓冲液充分洗膜3次，每次10 min，发光压片显色，以β-actin作为内参，Image J软件分析结果。

#### 1.2.7 统计学分析

所有实验重复3次，采用SPSS 20.0统计软件分析数据。数据均用Mean±SD表示，统计学方法采用方差分析，两独立样本均值比较采用*t*检验，*P* < 0.05认为差异有统计学意义。

## 2 结果

### 2.1 RNA质检、芯片和RT-PCR结果

提取RNA后，用紫外分光光度计结果显示A260/A280分别为2.03和2.01，表明所提取的总RNA纯度较高。RNA变性琼脂糖凝胶电泳结果显示28S和18S核糖体RNA亮而浓，28S带密度大约是18S带的2倍，表明提取的总RNA完整性好（图 1A）。质检结果表明，总RNA样品符合芯片质量要求。芯片结果及RT-PCR验证结果显示，107条miRNA差异表达（差异倍数≥2倍）于A549及其顺铂耐药株A549/DDP，其中miR-192是在A549/DDP细胞株中表达上调最明显的miRNA之一（表达量是A549细胞的99.01倍）（图 1B）。采用RT-PCR验证miRNA芯片结果显示，A549/DDP细胞株中miR-192表达量是A549细胞株表达量的37.59±0.35倍（*P* < 0.05）。miR-192转染细胞后，RT-PCR检测转染效率结果显示，在转染了miR-192 mimic的A549细胞与NC组比较，miR-192表达水平提高到234.21±5.45倍（*P* < 0.05）；同时，在转染了miR-192 inhibitor的A549/DDP细胞与NC组比较，miR-192表达水平减低了0.025±0.0025倍（*P* < 0.05）。

**1:**
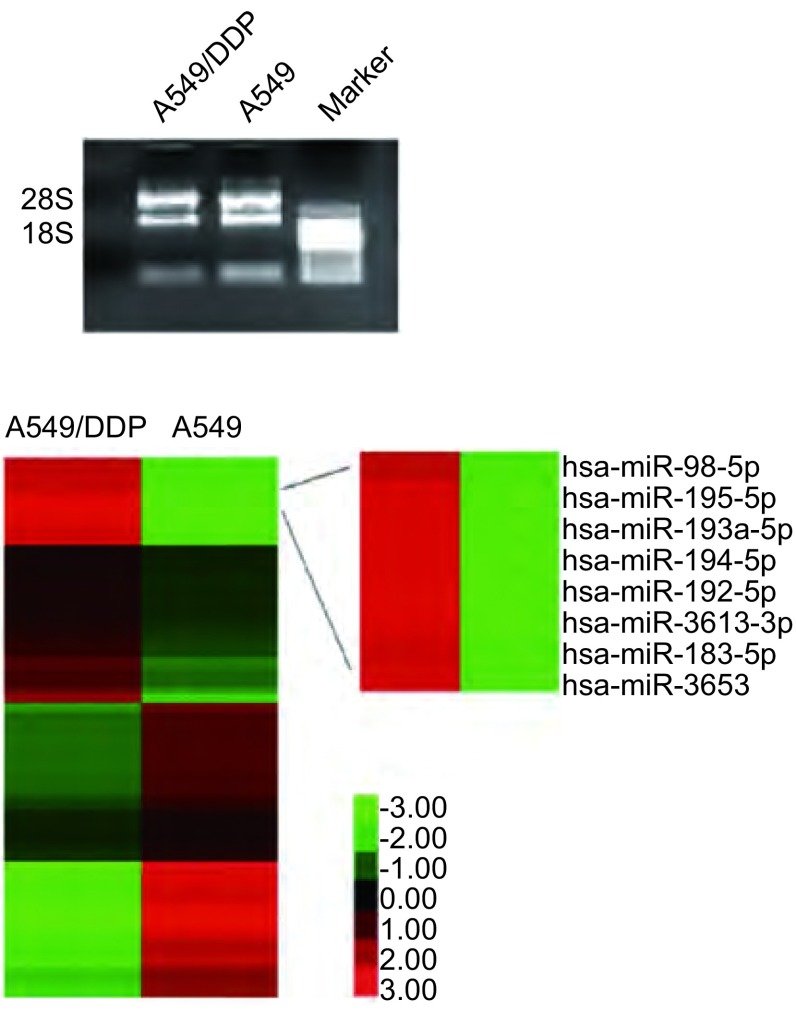
RNA质检后应用miRNA芯片检测结果。A：琼脂凝胶电泳检测RNA完整性；B：A549和A549/DDP中差异表达的miRNAs部分芯片结果。 Quality tested RNA was measured by miRNA microarray. A: The integrity of RNA was examined with agarose gel electrophoresis; B: Part of differentially expressed miRNAs between A549 and A549/DDP cells were listed as above.

### 2.2 MiR-192转染前后细胞IC_50_结果

A549细胞转染miR -192 mimic后，顺铂作用于细胞的IC_50_值为（15.22±0.91）μg /mL，较转染NC组IC_50_[（3.42±0.90）μg /mL]明显提高（*P* < 0.05）；而转染了miR-192 inhibitor的A549/DDP细胞，顺铂作用于细胞的IC_50_值为（7.28±1.10）μg/mL，较转染NC组IC_50_[（12.51±1.00）μg /mL）明显降低（*P* < 0.05）（图 2）。结果表明，miR-192的过表达使细胞对顺铂的耐受性提高，miR-192可以诱导细胞对顺铂的耐药。

**2:**
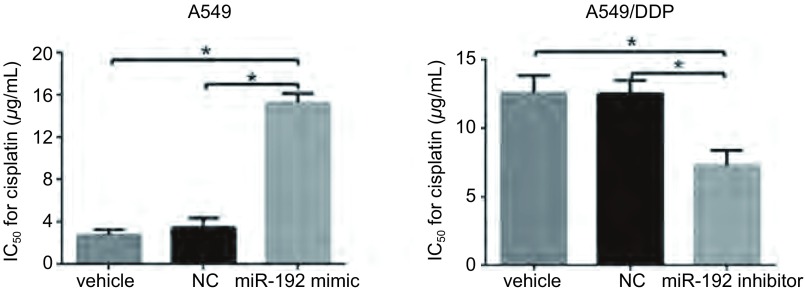
CCK-8检测细胞转染后IC_50_结果。^*^P < 0.05。 Half inhibition concentration (IC_50_) or cisplatin of cells after transfection was measured by CCK-8 assay. ^*^P < 0.05; NC: negative control.

### 2.3 MiR-192转染对细胞凋亡的影响

流式细胞术结果显示，转染miR-192 mimic的A549细胞凋亡率为（6.45±0.87）%，转染miR-NC组的细胞凋亡率为（27.52±0.85）%，差异具有统计学意义（*P* < 0.05）；转染miR-192 inhibitor的A549/DDP细胞凋亡率为（44.46±0.70）%，而转染miR-NC组的细胞凋亡率为（31.41±4.95）%，差异具有统计学意义（*P* < 0.05）。结果表明miR-192过表达可以减少由顺铂作用而引起的细胞凋亡（图 3）。

**3:**
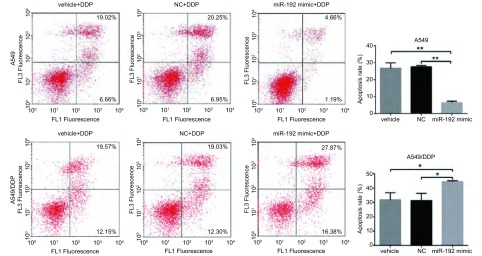
流式细胞术测细胞转染后凋亡结果。^*^P < 0.05；^**^P < 0.01。 Apoptosis rate of cells after transfection was detected by FCM assay. ^*^P < 0.05; ^**^P < 0.01.

### 2.4 双荧光素酶报告基因结果

在共转染了Bim 3’-UTR双荧光素酶报告基因载体和miR-192 mimic的A549细胞中，检测到细胞中荧光素酶活性显著下调，而在共转染了Bim 3’-UTR双荧光素酶报告基因载体和miR-192 inhibitor的A549/DDP细胞中，荧光素酶活性显著上调。同时，当miR-192 mimic或miR-192 inhibitor与mutant-Bim 3’-UTR双荧光素酶报告基因载体共转染细胞时，没有观察到荧光素酶活性的改变。这些结果表明，miR-192在肺腺癌细胞中通过靶向作用于3’-UTR区负向调节*Bim*基因的表达（图 4）。

**4:**
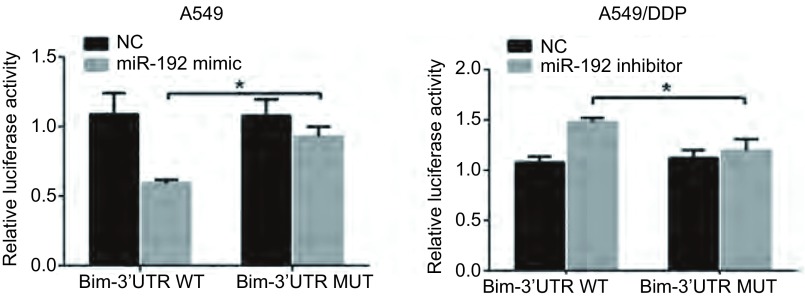
双荧光素酶报告基因结果。^*^P < 0.05。 Dual luciferase reporter assays in A549 and A549/DDP cells. ^*^P < 0.05.

### 2.5 MiR-192对靶基因*Bim*表达的影响

RT-PCR结果显示，转染miR-192后，细胞内*Bim*基因mRNA表达水平无明显改变。Western blot结果显示，相较于转染NC的细胞，转染miR-192 mimic的A549细胞中Bim蛋白表达下调；相较于转染NC的细胞，转染miR-192 inhibitor的A549/DDP细胞中Bim蛋白表达上调（图 5）。此结果表明，miR-192通过转录后水平负向调控Bim的表达而发挥作用。

**5:**
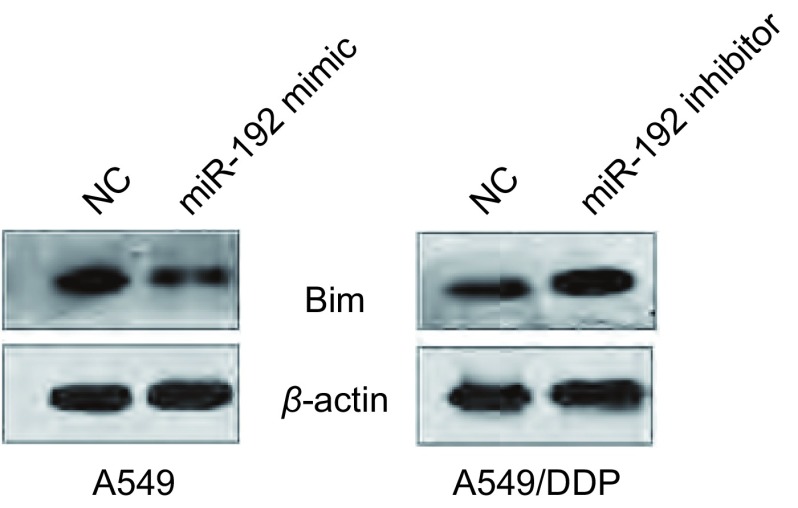
Western blot检测Bim蛋白表达结果 The protein level of Bim was analyzed by Western blot

## 3 讨论

MiRNA芯片技术是一种快速有效分析miRNA表达谱的方法，具有较高灵敏度和特异性。结合RT-PCR检测, 能基本准确地找到A549及其耐药细胞株A549/DDP细胞中差异表达的miRNAs。研究^[5]^报道miRNAs差异表达于不同的耐药肿瘤细胞中，发挥着诱导或者逆转肿瘤细胞耐药的功能。miR-34a的过表达可以靶向负性调节*SIRT1*基因的表达而诱导细胞凋亡，从而逆转前列腺癌细胞对喜树碱类化疗药物的耐药。MiR-200c在食管癌细胞中可以调节Akt通路从而影响癌细胞在顺铂作用下的细胞凋亡和耐药性^[6]^。我们的实验结果表明miR-192显著高表达于肺腺癌顺铂耐药株A549/DDP细胞中，我们推测miR-192可能与肺癌顺铂耐药有关。有研究报道了miR-192具有调节细胞生长，细胞周期、细胞凋亡等生物学功能，它的差异表达可以用来辅助临床肿瘤诊断、分期、侵袭、转移和预后等^[7-10]^。此外，miR-192可以通过调节P53而影响细胞的周期分布从而影响结直肠癌细胞对5-Fu的耐药^[11]^。本研究结果表明miR-192的过表达可以抑制顺铂作用于细胞而引起的细胞凋亡、诱导肺癌细胞对顺铂的耐药。为了进一步探索miR-192诱导肺癌细胞顺铂耐药的机制，我们通过生物软件预测和双荧光素酶报告基因法找到了*Bim*为miR-192的靶基因之一。*Bim*为Bcl2家族的一员，是一个促凋亡基因。Bcl-2家族蛋白是细胞凋亡过程中最重要的调控因子，根据它们对细胞凋亡调控的作用可分为两大类：一类是抗凋亡蛋白，如Bcl-2、Bcl-xl、Bcl-w和Mcl-1等，大多数成员含有四个BH（Bcl-2 homology domain）结构域，即有BH1-BH4；第二类则是促细胞凋亡蛋白，如Bax、Bak等，通常含有三个结构域（即BH1-BH3）。在促凋亡蛋白中，有些分子仅含有一个BH3结构域，如Bim、Bid、Bad、Bike等，BH3结构域是它们的核心功能区。Bim可促进许多肿瘤细胞的死亡，如肺癌、乳腺癌、骨肉瘤和黑色素瘤细胞等，很多化疗药物通过调节Bim表达来发挥杀灭肿瘤细胞的功能^[12]^。Bim的表达水平与顺铂耐药相关，研究^[13]^报道在卵巢癌顺铂敏感细胞株中通过促进Bim蛋白的降解诱导细胞对顺铂的耐药。随着对miRNA的深入研究的发展，有研究报道了miRNA可以通过靶向作用于Bim来调节癌细胞的生物学行为，Romano等^[14]^报道了在非小细胞肺癌细胞中miR-494可以通过靶向下调Bim的表达而调节TRAIL诱导的细胞凋亡。Yan等^[15]^发现抑制miR-17-5p的表达，其靶基因*Bim*蛋白表达水平上调，从而导致胰腺癌细胞对吉西他滨的化疗耐药。我们的实验结果表明，miR-192可以靶向作用于*Bim*基因的3’-UTR区，并负向调节Bim的表达，导致肺腺癌细胞在顺铂作用下的细胞凋亡率降低。

本研究表明，miR-192通过靶向负性调节促凋亡基因*Bim*的表达，减少肺癌细胞在顺铂作用下的细胞凋亡率，从而诱导肺癌细胞对顺铂的耐药，阐明了肺癌顺铂耐药性产生的机制之一，提示miR-192可以作为抑制肺癌顺铂耐药的靶点，抑制肺癌细胞内miR-192的表达可能是在未来临床治疗中逆转肺癌顺铂耐药的一种新方法。
